# Thermo-Optic Phase Shifter with Interleaved Suspended Design for Power Efficiency and Speed Adjustment

**DOI:** 10.3390/mi13111925

**Published:** 2022-11-08

**Authors:** Feng Gao, Wu Xie, James You Sian Tan, Chew Ping Leong, Chao Li, Xianshu Luo, Guo-Qiang Lo

**Affiliations:** Advanced Micro Foundry Pte Ltd., 11 Science Park Road, Singapore 117685, Singapore

**Keywords:** silicon photonics, thermo-optic phase shifter, photonic-integrated circuits

## Abstract

Conventional thermo-optic devices—which can be broadly categorized to that with and without a thermal isolation trench—typically come with a tradeoff between thermal tuning efficiency and tuning speed. Here, we propose a method that allows us to directly define the tradeoff using a specially designed thermo-optic phase shifter with an interleaved isolation trench. With the design, the tuning efficiency and speed can be precisely tailored simply by controlling the duty ratio (suspended length over total heater length) of the suspended design. Phase shifters are one of the main components in photonic-integrated circuits, and having phase shifters with a flexible design approach may enable the wide adoption of photonic applications such as an optical neural network and LiDAR.

## 1. Introduction

Silicon photonics is one key technology that provides high-density, large-scale, and compact on-chip optical systems that can be mass produced using mature complementary-metal-oxide-semiconductor (CMOS) technology [1,2]. Such a platform is already applied to many complex photonic-integrated circuits, such as quantum optical computing [3,4,5], LiDAR [6,7,8], data communications [9,10,11], sensing [12,13], and optical neural networks [14,15,16,17]. Among above applications, the thermo-optic phase shifter is one of the key building blocks. 

The common thermo-optic phase shifter design includes the use of a metal heater on the top of an optical waveguide [18]. To improve phase-shift modulation efficiency, a thermal isolation structure (in the form of ‘suspended design’) is introduced, which is formed by removing the silicon substrate under the waveguide region [19,20]. The air isolation region formed as a result of removing the silicon structure results in stronger heat confinement within a small waveguide volume which can significantly enhance the thermal tuning efficiency. However, this comes with a tradeoff. Due to stronger heat confinement, thermal dissipation is less effective, which in turn results in a lower tuning speed. Table 1 summarizes the performance comparison of several representative designs. While a conventional thermo-optic phase shifter design results in a higher tuning speed and lower tuning efficiency, the suspended thermo-optic phase shifter design is the opposite—a high tuning efficiency but low tuning speed. 

In this work, we propose a thermo-optic design that enables us to define the tradeoff. In the design, we introduce interleaved isolation trenches to enable the convenient control of thermal conductance—which effectively enables the flexible control of both the tuning efficiency and speed. This is made possible by controlling the ratio of isolation trench length over heater length (hereafter, suspended design duty ratio). With such control, specific phase shifter requirements (which differs from application to application) can be readily met.

## 2. Theory

The mechanism by which thermo-optic phase shifters shift the optical phase of an optical signal is as follows: a change in temperature in optical waveguide induced by a heater placed in close proximity to the waveguide results in a shift in the waveguide refractive index, therefore, the optical phase of the optical signal traverses through the waveguide. The two main performance metrics of a thermo-optic phase shifter is the thermal tuning efficiency and response time. Thermal tuning efficiency is defined by the electrical power *P_π_* required to achieve π-phase shift. Here, a high efficiency phase shifter would need a lower electrical power. Assuming there is no spatial gap or finite thermal conductance between the heater and optical waveguide, *P_π_* can be defined as

*P_π_* = *G* × *A* × Δ*T_π_* (1)

where *G* is the thermal conductance of an optical waveguide to its surrounding region (for example, Si substrate), *A* is the effective area traversed by heat (which is related to heater width *W* and heater length *L*), and Δ*T_π_* is the temperature change to achieve π-phase shift (which is related to the operation wavelength and thermo-optic coefficient) [18,21].

The thermo-optic tuning response time constant *τ*, on the other hand, is the time required by the thermo-optic phase shifter to shift the optical phase. *τ* is inversely proportional to the thermal tuning speed, and can be defined as:

*τ* = *H*/(*G* × *A*) (2)

where *H* is the heat capacity of the heated optical waveguide (of one of the MZI arms). *τ* can be retrieved from rise time (*T_rise_*) and fall time (*T_fall_*), from which the time taken for the voltage level to rise from 10% to 90% (*T_rise_*) and fall from 90% to 10% (*T_fall_*) can be precisely determined on the rising and falling edges of the signal, respectively, using the relation [18]:

*T_rise_* (*T_fall_*) ≅ 2.197 × *τ* (3)

It is evident from Equations (1)–(3) that both the thermal tuning efficiency and tuning response time are directly related to thermal conductance *G*. Because *G* of the air is almost three orders of magnitude lower than that of silicon and silica, a straightforward method to reduce *G* (which increases the tuning efficiency but reduces the tuning speed) is obtained by isolating the area surrounding the heater [22,23]. In this work, we extend the concept further by adjusting the length of the suspended structure (or ‘interleaved’ suspended structure) to precisely control both the tuning efficiency and tuning speed by changing the length of the isolation trench which changes *G*. An area with a suspended design has ultra-low *G*, which could reduce *G* of the whole phase shifter area. This enables us to directly define the tuning efficiency and tuning speed tradeoff, as we shall thoroughly discuss.

## 3. Structures and Fabrication

The length of the interleaved suspended structure, which can be arbitrarily set to modify *G*, can be systematically characterized by introducing the term duty ratio *D*, defined by the ratio of suspended length *L_suspended_* over total length *L_total_*:

*D* = *L_suspended_*/*L_total_* × 100% (4)

**Figure 1:**
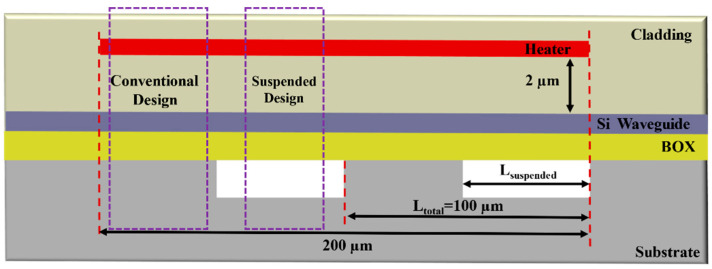
Section view of thermo-optic phase shifter with interleaved suspended design.

It is clear that *D* = 0% and *D* = 100%, respectively, imply conventional design without the suspended structure and fully suspended design, respectively. In this work, by fixing the total heater length area at 200 µm (which includes two interleaved area with *L_total_* of 100 µm each), as shown in Figure 1, we can vary *D* = 0% to *D* = 100% by correspondingly changing *L_suspended_* from 0 µm to 100 µm.

**Figure 2:**
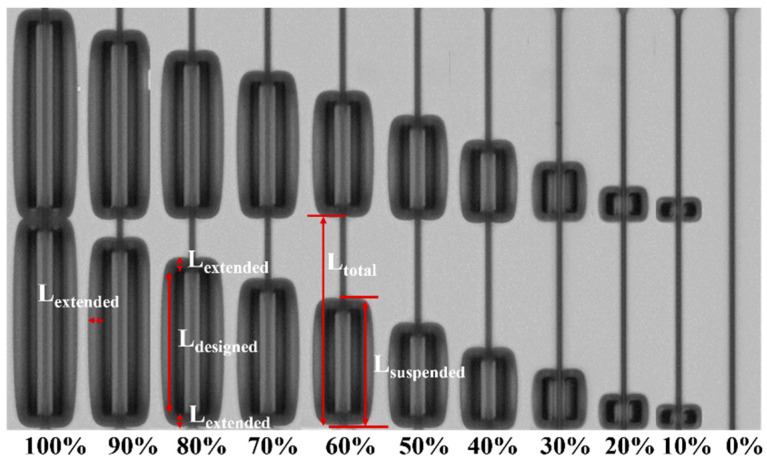
Top-view optical microscopy image of the fabricated thermo-optic phase shifters with interleaved isolation trenches with different duty ratios.

We fabricated the devices on the SOI wafers of the 220 nm-thick top Si layer and 3-µm-thick BOX layer wafers on the Advanced Micro Foundry (AMF) standard platform. The TiN metal layer, acting as a heater layer, is deposited and formed on top of the Si waveguide with a 2 µm vertical distance. The thickness of oxide on the TiN metal heater is approximately 1 µm. The suspended area is formed by isotropic etching to partially remove the Si substrate beneath the Si waveguide. Figure 2 shows the top-view optical microscopy image of the fabricated thermo-optic phase shifters with interleaved isolation trenches with different *D*. The actual length and width of the suspended design is slightly larger than the designed length due to the extension of the isotropic etching. The actual length of the suspended design *L_suspended_* is calculated as,

*L_suspended_* = *L_designed_* + 2 × *L_extended_* (5)

where *L_designed_* is the designed length. *L_extended_* is the extended length, which is caused by isotropic etching. *L_extended_* is related to the isotropic process, which is approximately 6 µm in the AMF standard process flow with a 5 µm-wide-designed oxide trench. Additionally, the thickness of the removed silicon substrate below the buried oxide in this work is approximately 15 µm. In this work, the length of suspended design *L_suspended_* includes the extended length *L_extended_*. To ensure that the extended length *L_extended_* is the same, all measured devices are from the same die of the same wafer.

## 4. Characterization and Results

**Figure 3:**
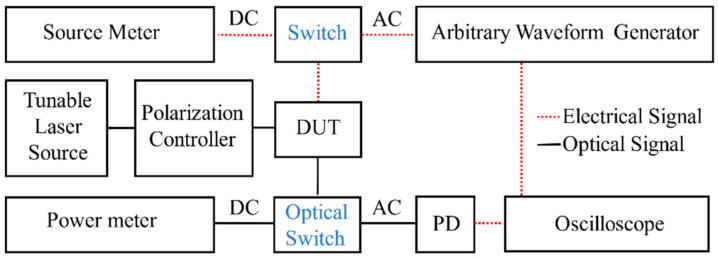
The block diagram of the measurement setup for DC and AC characterization. DUT: Device under test. PD: Photodetector.

To measure the performance of the thermo-optic phase shifter with interleaved isolation trenches, we designed the thermo-optic phase shifter such that it is seamlessly placed on one of the waveguide arms of an unbalanced Mach–Zehnder interferometer (MZI). The metal heater of the phase shifter introduces heat to induce the required optical phase shift. Inverse tapers are used to couple the optical signal to and from the MZI. The MZI comprises two 1 × 2 multi-mode interferometers (MMI) that split and combine the optical signal, respectively, to and from two 500 nm-width single-mode Si waveguides with different lengths. To measure the thermal tuning efficiency, we used the direct current (DC) characterization setup, as illustrated in Figure 3. In the setup, lensed fiber is used to couple the polarized quasi-TE light of 1550 nm wavelength into the device via an inverse taper. An electrical probe is placed onto the aluminum pads of the device to apply voltages from a source meter. The current and output optical power are measured by a source meter and power meter, respectively. Then, the electrical power consumption to achieve *π* phase shift *P_π_* could be extracted, based on the applied voltage, measured current, and measured optical power. To measure the thermal tuning response, we used the alternating current (AC) characterization setup, which is similar to the DC characterization setup, except that the output optical signal from the MZI is sent to the external photodetector (which converts the optical signal to the electrical signal), and the square electrical drive signal generated by an arbitrary waveform generator is split into two paths, i.e., to the heater via an electrical probe, and to the oscilloscope as reference. The signal rise time *T_rise_* and fall time *T_fall_* could be directly determined from the oscilloscope.

**Figure 4:**
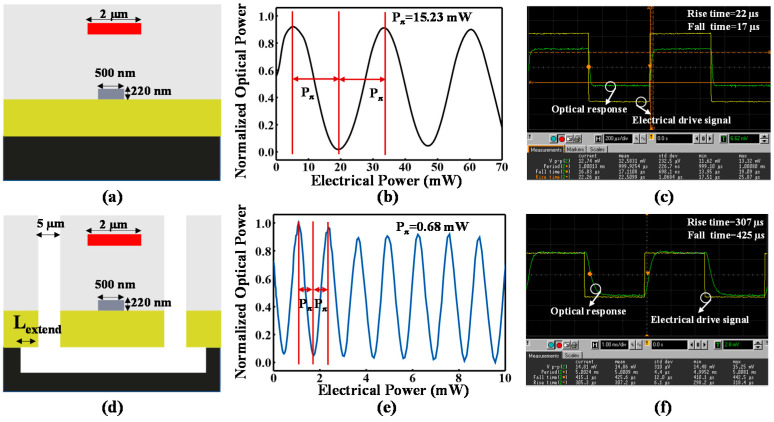
Measured thermal tuning performance of the conventional design with duty ratio *D* of 0% and fully suspended design with duty ratio *D* of 100% at 1550 nm wavelength. (**a**) Cross-section of conventional design. (**b**) DC characterization of the conventional design. (**c**) AC characterization of conventional design. (**d**) Cross-section of the suspended design. (**e**) DC characterization of the suspended design. (**f**) AC characterization of the suspended design.

Figure 4a shows the cross-section of the conventional design with the duty ratio *D* of 0. An additional 5-µm trench is introduced for a fully suspended design with the duty ratio *D* of 100%, as shown in Figure 4d, The electrical power consumption *P_π_* of the suspended design is 0.68 mW, which is one order of magnitude smaller than that of the conventional design, as shown in Figure 4b,e. Owing to the tight heat confinement in the optical waveguide area, the thermal tuning efficiency improves significantly. However, due to the poor heat dissipation, the rise time of the suspended design increases from 22 µs to 307 µs, and the fall time increases from to 17 µs to 425 µs, as shown in Figure 4c,f, thus, indicating that the thermal tuning response time increases by one order of magnitude.

**Figure 5:**
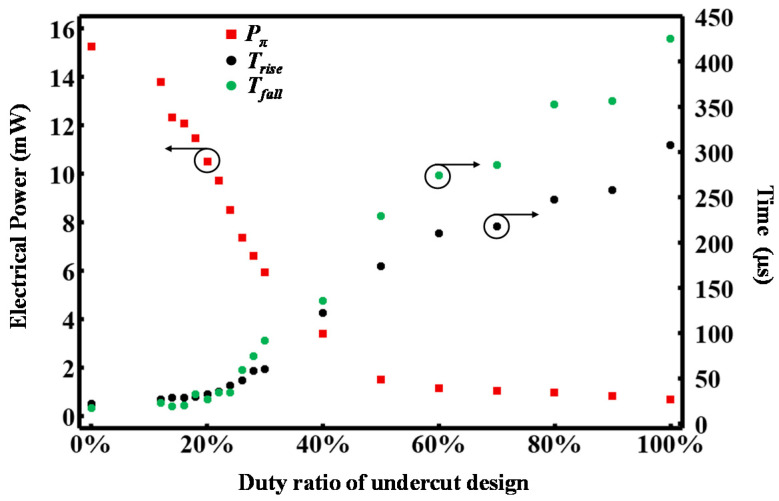
Summarized thermal tuning performance for the phase shifters with a different duty ratio *D*, including the electrical power consumption *P_π_* (red-dotted), rise time *T_rise_* (black-dotted) and fall time *T_fall_* (green-dotted).

Figure 5 summarizes the measured thermal tuning performance of the thermal phase shifters (*P_π_*; *T_rise_* and *T_fall_*—from which *τ* can be retrieved) with respect to *D* at the 1550 nm optical signal wavelength. As *D* is increased from 0% to 100%, *P_π_* decreases from 15.23 mW to 0.68 mW, while both *T_rise_* and *T_fall_*, respectively, increase from 22 µs to 307 µs and from 17 µs to 425 µs. The results conform to Equations (2) and (3). With such a controllable physical parameter in the form of *D*, both the thermal tuning efficiency and speed can be precisely tailored to conveniently meet the specific requirements for different applications. 

## 5. Conclusions

In summary, we propose and demonstrate for the first time a thermo-optic phase shifter with interleaved suspended structures. Our specially designed phase shifter enables the convenient control of both the tuning efficiency and tuning speed. This is achieved via our customized duty ratio of the interleaved suspended structure. Our design provides flexibility to finely tune the required thermal efficiency and tuning speed, which can broadly differ depending on applications such as quantum optical computing, optical neural network, and LiDAR.

## Figures and Tables

**Table 1:** Performance comparison of thermo-optic phase shifter on Si channel waveguide.

Heater Material	Structure	Heat Isolation	Tuningv Efficiency	Switch Time	Ref.
TiN	MZI	No	21.4 mW	12 µs	[18]
TiN	MZI	Suspended	0.49 mW	144 µs	[19]
TiN	Ring	Suspended	2.4 mW	170 µs	[20]

## Data Availability

Not applicable.
